# A retrospective cross-sectional study of Irish national dairy calf mortality data; 2016–2020

**DOI:** 10.1186/s13620-025-00316-0

**Published:** 2025-11-25

**Authors:** Lisa Buckley, Aideen Kennedy, Maresa Sheehan, Mícheál Casey, Rob Doyle, Elizabeth A. Lane

**Affiliations:** 1 Kilkenny Regional Veterinary Laboratory, Department of Agriculture Food and the Marine, Kilkenny, Ireland; 2 Department of Agriculture, Food and the Marine, Backweston Campus, Kildare, Ireland

**Keywords:** Calf mortality, Calf welfare, Dairy herd, Herd expansion, Ireland

## Abstract

**Background:**

Since the abolition of EU milk quotas in 2015, the Irish dairy industry has expanded with a 16.6% increase in calf births since 2011. Calf losses have major implications for the economic viability and sustainability of dairy enterprises. There is a paucity of literature on mortality in calves from birth to six months, particularly at a national herd level. Previous studies have tended to focus on herd size as a risk factor for calf mortality rather than the possible influence of herd expansion. The purpose of this study is to quantify any association between dairy herd expansion and the risk of being classified as a high or low calf mortality herd based on analysis of national dairy herd identification and movement records in Ireland from 2016–2020.

**Results:**

In calves aged under 6 months, herds that expanded > 20% (OR 1.23 95% CI: 1.10–1.37, *p* < 0.001) and > 45% (OR 1.22, 95% CI:1.09–1.36, *p* = 0.001), were more likely to have > 10% herd calf mortality, compared to herds that did not increase in size. Newly established herds were more likely to have poor mortality outcomes (OR 2.44, 95% CI: 1.82–3.29, *p* < 0.001) compared to herds that did not increase in size. Herd expansion < 20% was not associated with mortality outcome. Herd ordinal location and herd size were significant risk factors for > 10% herd calf mortality.

**Conclusion:**

This study has demonstrated that herds that have expanded > 20% over five years and newly established herds were more likely to have poor mortality outcomes. It suggests that new entrants into dairy farming may benefit from targeted emphasis on herd health management. The results also highlight the value of national data as a tool to determine optimisation of farm interventions and surveillance and policy decisions to prioritise animal health and welfare.

## Introduction

The dairy industry worldwide has transformed in the past few decades. Over this period, world milk production has increased by 60% and is expected to grow by 1.7% per annum over the next decade [1]. Dairy farmers are under increasing scrutiny from consumers and regulatory authorities to achieve higher standards in food safety, biosecurity, and animal welfare [2]. The concepts of ‘One Health’ and antimicrobial resistance [3], as well as the reduction of greenhouse gas emissions [4], demand the achievement of the highest standards by dairy farmers. Consumer expectations continue to evolve and labelling of food to assure welfare standards are upheld, may become compulsory in the near future [5].

### Dairy herd expansion in Ireland

Ireland too has experienced an increase in scale of dairy production since milk quota abolition in 2015. A survey of Irish dairy farmers found that 84% of respondents had increased their herd size [6]. The number of dairy cows on Irish farms has increased by 35% in the period 2013–2020 and in 2020 there were approximately 1.5 million dairy cows in Ireland [7]. Despite this increase in cow numbers, herd numbers are down 6% since 2011, while average herd size has increased to 90 cows, which is an increase of 53 compared to the year 2000 [8]. This expansion in cow numbers also brings expansion in calf numbers. Department of Agriculture, Food and the Marine (DAFM) statistics [9], indicate calf births have been steadily increasing, 16.6% since 2011 with the biggest growth in 2015, when EU milk quota abolition occurred, with 6% more calves born compared to 2014. The report also specifies there were 1.3million dairy calves registered in Ireland in 2016 and this rose to 1.5million in 2020. Therefore, increasing numbers of dairy cattle are becoming concentrated in a decreasing number of herds. As stocking densities on farms increase, the risk of spread of infectious disease increase, close contact between animals is facilitated allowing contagious pathogens e.g. BVD to spread more easily [10]. Farm labour also comes under strain, which in turn, can endanger animal welfare [11].

### Calf mortality

Calf losses have major implications for the economic viability and sustainability of dairy enterprises [12]. Calf health was identified as one of the most important issues facing the Irish livestock industry in an expert Policy Delphi study in 2010 [13]. All aspects of calf health, including mortality rates, needs to be continuously monitored and reviewed.

Calf mortality studies carried out in Ireland, [14–19], in Europe, [20–31], in the US, [32, 33], in Africa, [34], in Asia, [35, 36] and New Zealand, [37], have reported variable mortality rates ranging from 3.1 to 10% in calves aged under 6 months. Comparisons between studies and countries are difficult due to the lack of a standardised definition of calf mortality [38, 39]. Different age classes and metrics are used to describe calf mortality in the literature. Some countries report mortality rates in terms of calf months on farm [28], while others focus on mortality rates over specific time periods [38]. There is also a paucity of published national herd level data on calf mortality in many countries with the exception of Hyde et al. [24]and Cooke and Wathes [22] in the UK, and Buttigieg et al. [21] in Malta and Gozo. Other studies have been conducted on a small number of animals or herds, that were followed over a short time period [23, 29, 40, 41].

Previous literature has identified many possible risk factors for calf mortality. Significant farm level risk factors include, herd size [17, 21, 23, 26, 28, 33, 37, 42], housing strategy [23, 26, 30, 31], region [37], number of animal purchases [26, 27, 41], season [19, 23, 24, 27, 35, 37, 42, 43] and morbidity [25, 37]. Significant animal level risk factors include, gender [17, 21, 24, 27, 37, 42, 43], twin calves [19, 20, 23, 43], parity of dam [18, 20, 43], breed [17, 24, 29, 30], dystocia at birth [18, 20, 23, 35, 43] and nutrition [25, 31]. The possible influence of herd expansion on calf mortality has not been well documented in the literature.

### Aim and objectives

The aim of this study was to quantify the association between dairy herd expansion (five levels) and the likelihood of being classified as a high- or low-calf mortality herd in calves aged under 6 months, based on analysis of all dairy herd mortality data in Ireland from 2016–2020. The main objective of the study was to investigate if calf mortality is higher in herds that have expanded compared to those that have not expanded and if mortality level varies depending on the level of expansion. Secondary objectives were to investigate the role of herd location, herd size, farm movements and Bovine Viral Diarrhoea (BVD) status in explaining the association between calf mortality and herd expansion.

## Materials and methods

### Study design and population

This study was a retrospective cross-sectional analytical study of calf mortality levels in the national dairy herd in Ireland from the years 2016–2020. Data was analysed from all registered dairy cattle farms. Irelands dairy industry is predominately a grass-based seasonal calving enterprise, with animals grazing outdoors on pasture for up to 9 months of the year [44].

### Data preparation

A data set of animal demographic records collected over a five-year period was obtained from the Animal Identification and Movement (AIM) database within the Department of Agriculture, Food and the Marine (DAFM) in Ireland. A total of 16,636, 16,663, 16,680 and 16,697 records from dairy herds, from the years 2016–2020, were available for analysis. The AIM data set also provided herd-level data on herd size, herd location, number of cattle movements onto the farm (from other farms and from marts), BVD infection status of the herd as well as calf mortality.

Final datasets and variables selected for analysis were first downloaded onto Microsoft Excel (https://office.microsoft.com/excel), arranged, checked for errors and missing values. Herds were anonymized and given unique identification numbers. Data was then imported into the Stata 16 software package (Stata LLC https://www.stata.com/), for data management and analysis. All dairy herds registered as a milk supplier were eligible for inclusion in the present study. Herds were excluded if zero births were recorded in any of the years examined 2016–2020, if herd size reduced over the study period or if the average number of births in the years 2019/2020 was less than ten. There were no missing values noted in the dataset.

### Power calculations

Calculations were carried out in Stata for a study power of 80% and 5% significance level. The sample size required to detect a minimum 1.2-fold increase in mortality rate associated with expansion, compared to herds that did not expand was 6,794 i.e., 3,397 in expanded and unexpanded groups. The study was adequately powered to detect this, with records from 16,146 dairy herds included in the dataset.

### Analysis

The unit of analysis was the herd. Calf mortality data was collated for each herd from the years 2016–2020 to obtain an average five-year mortality proportion. A total of 16,146 animal identification and movement records were selected for inclusion in the study to investigate the role of dairy herd expansion on calf mortality level. Table 1 summarizes the characteristics of the studied variables among dairy cattle herds in Ireland.

**Table 1:** Showing the characteristics of the studied variables and their categories among dairy cattle herds in Ireland (*n* = 16,146)

Variable	Category	Number	%
Outcome			
Average mortality in up to six-month-old calves	< 10%	12,637	78.27
	> 10%	3509	21.73
Exposure			
Herd expansion percentage	−5 to + 3	3983	24.67
	+ 3 to 20	3986	24.69
	+ 20 to 45	3981	24.66
	+ 45 to 1091	3985	24.68
	––––-	211	1.31
Potential confounder			
Herd location	South-east	5122	31.72
	South-west	7145	44.25
	North-west	1394	8.63
	North-east	2485	15.39
Herd size	0–90	4074	25.23
	91–140	4001	24.78
	141–214	4057	25.13
	215–1742	4014	24.86
Farm to farm moves	No moves	1340	8.30
	Any move	14,806	91.70
Mart to farm moves	No move	2958	18.32
	Any move	13,188	81.68
All movements	0–11	4233	26.22
	12–34	3874	23.99
	35–97	4012	24.85
	98–10,065	4027	24.94
BVD status	Negative	15,599	96.61
	Positive	547	3.39

#### Exposure

The exposure of interest in this study was herd expansion percentage. This was calculated as the average number of herd births registered in the years 2019 and 2020 divided by the average number of births registered in the years 2013 and 2014, to generate an expansion percentage for each herd. These years were chosen to capture any expansion in herd numbers that may have occurred after the EU milk quota abolition in 2015. Herd expansion was then categorised into four levels based on the quartiles of the distribution of herd expansion. Level one, the referent level, represents herds that stayed the same size while levels two, three and four represents up to 20%, 45% and 1091% expansion in herd size respectively. Level five represents newly breeding herds. A newly breeding herd was defined as herds with greater than ten births registered in 2019/2020, with no births registered in 2013/2014.

#### Outcome

The outcome of interest in this study was herd calf mortality classification, which was reported at the time point from birth to 6 months. Within DAFM calf mortality is defined as the number of calves registered as dead by a designated age divided by the number of births. This sum was tabulated and collated from each herd over a five-year period for established herds (2016–2020) and over a two-year period for newly established herds (2019–2020), to produce an average herd calf mortality ratio at 6 months. This continuous variable was recoded to generate a binary outcome variable to classify herds into high and low mortality categories. A value of 10% was chosen as the marker for division into high and low mortality herds based on results from previous studies [14, 15, 24].

#### Covariates

Herd location [6, 23, 28], herd size [18, 23, 26], movement of animals [27, 30, 41] and recent herd BVD status [45], were considered as potential confounders based on previous research. These variables were regrouped for descriptive and interpretative purposes as shown in Table 1. All potential covariates were tested for collinearity. A Pearson’s correlation coefficient value > 0.5 was considered evidence of collinearity.

### Analysis plan and steps

#### Descriptive analysis

Cross tabulation of all variables was performed to assess differences in baseline characteristics between herds with high and low mortality. For each variable, the number and percentage distribution in the two mortality groups (high and low) was presented. Chi-squared tests were used to calculate a crude measure of association (odds ratios) between the primary exposure (herd expansion percentage) and other covariates (potential confounders) with the outcome. Chi-squared tests were also used to investigate associations between the primary exposure (herd expansion percentage) and other potential confounders. A cut-off *p*-value of 0.1 was chosen to retain all potentially useful covariates for the further analysis.

#### Mantel–Haenszel analysis and regression modelling

Independent variables recording *P* < 0.1 were included in a logistic regression model. A manual backwards elimination with a forward step was applied with significant variables (*P* < 0.05) retained in the final model. Separately adjusted measures of association between the exposure and outcome were reported for each potential confounder and the corresponding odds ratios were calculated. Three criteria were used to assess potential confounding, changing the strength of the association (odds ratio) between herd expansion and mortality level (by at least 10%) and a significant association with both herd expansion and mortality level. Effect modification was assessed by examining stratum specific odds ratios for each covariate. Herd size was considered an a priori* confounder*. Likelihood Ratio Tests (LRT) were used to test departures from linear effects for categorical variables**.**

Multivariable logistic regression was chosen over Cox regression as the dataset did not include time to event/mortality. Furthermore, odds ratios have been reported in previous literature, allowing for comparisons.

#### Sensitivity analysis

To assess the goodness of fit of the logistic regression models to the dataset, sensitivity and specificity tests for the outcomes of interest (high or low) herd mortality, were calculated and plotted creating a receiver operating characteristic (ROC) curve and the area under the curve (AUC) was calculated.

## Results

### Descriptive analysis

The final dataset included animal identification and movement records from 16,146 herds after excluding those records with zero number of births recorded (*n* = 666) and herds that reduced in size or had an average of < 10 births in 2019/2020 (*n* = 190). The median number of births recorded in 2016 was 68 (IQR: 43–101), this rose to 74 in 2020 (IQR: 45–114). The median percentage of calves lost under 6 months in 2016 was 6.12% and in 2020, was 7.02%.

#### Mortality

Most herds (78.27%) reported < 10% mortality in calves under 6 months over the study period. Table 2 shows the median and interquartile ranges of crude mortality percentages of the national dairy herd per year. Over half of herds (63%) achieved calf mortality levels < 8%, 48% of herds < 6% and 33% of herds < 4%.

**Table 2:** Crude mortality percentages (calf deaths to births) per year (2016–2020) for all dairy herds (median and interquartile range)

Year	No. of herds	Median (%)	Interquartile range (%)
2016	16,636	6.12	2.08–11.54
2017	16,677	5.36	1.75–10.35
2018	16,696	5.79	1.92–10.82
2019	16,686	4.55	1.38–9.10
2020	16,627	5.16	1.54–9.82

#### Herd expansion

The quartiles of the distribution of herd expansion percentage indicated 74.03% of herds in the dataset expanded to some degree, while 24.67% did not increase in size. The percentage increase in herd size ranged from 3 to 1091%. There were 1.31% or 211 newly established herds since 2016.

#### Herd location

**Fig. 1:**
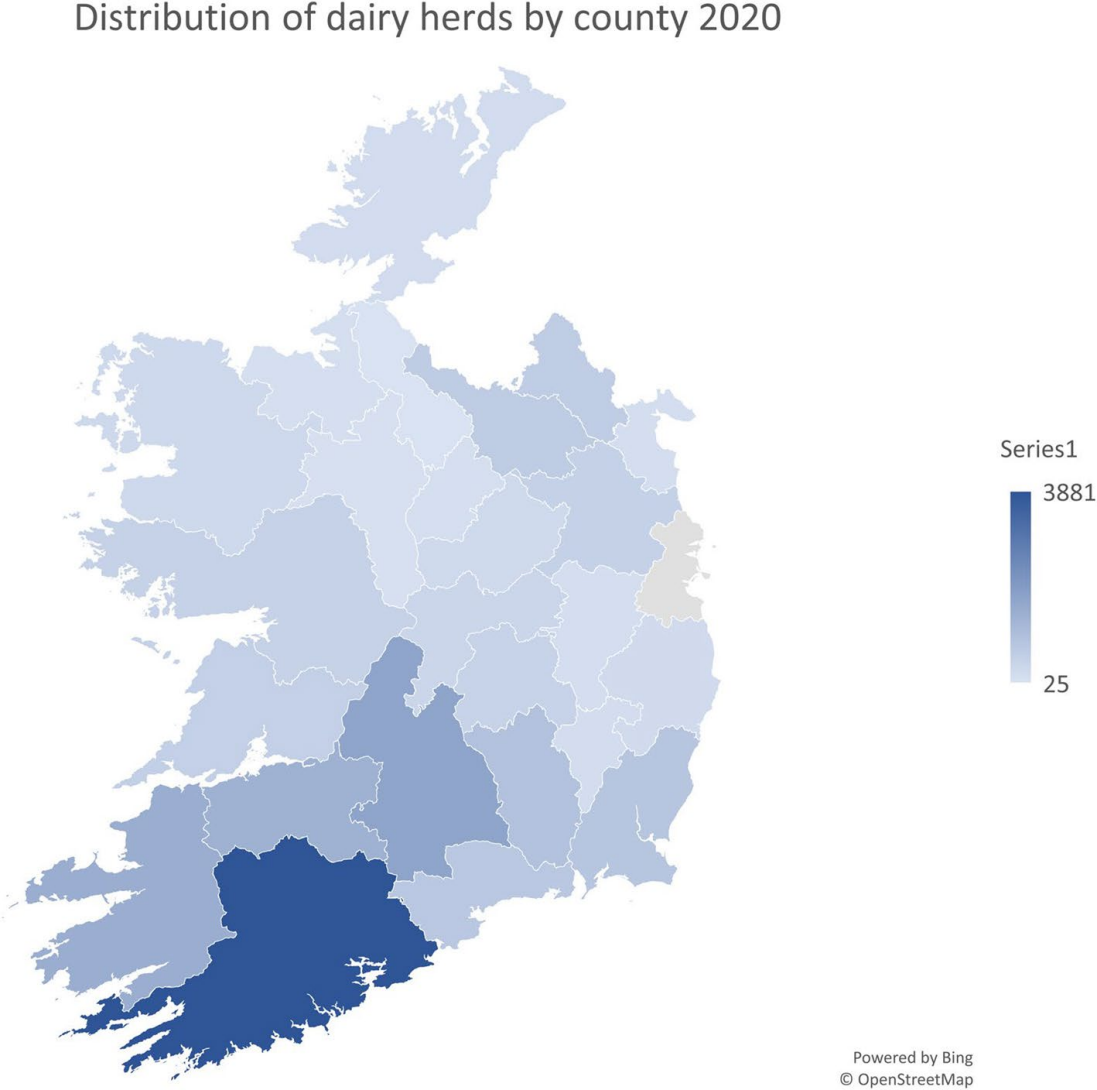
The distribution of dairy herds in Ireland in 2020

Figure 1 displays the dairy herd distribution in Ireland in 2020. The southwest of the country had the highest percentage of dairy herds (44.25%), followed by the southeast (31.72%), northeast (15.39%) and northwest (8.63%).

#### Herd size

The quartiles of the distribution of herd size ranged from a mean of 61 animals in the lowest quartile to a mean of 335 animals in the upper quartile.

#### Animal movements onto the farm

A small percentage of herds (8.30%) recorded no moves onto the farm from other farms. Similarly moves onto the farm from marts were also low (18.32%), during the five-year period in this study. The quartiles of the distribution of all moves onto farms ranged from a mean of 5 moves in the lowest quartile to a mean of 299 moves in the upper quartile.

#### BVD status

Five hundred forty-seven herds (3.39%) recorded a recent positive BVD test result in the years 2019/2020. Table 1 contains a description of all risk factors for calf mortality included in the analysis.

### Crude estimates

The distribution and associations of the univariate analyses between herd expansion and other herd level risk factors with calf mortality under six months are summarized in Table 3.

**Table 3:** Univariable association between herd expansion and other herd level risk factors for calf mortality from birth to 6 months of age

Risk Factors	Total (%) (*N* = 16,146)	Mortality < 10%	Mortality > 10%	OR (95% CI)	*P* value
Expansion Percentage					
1 (−5 + 3%)	3983 (24.67)	3213 (80.67)	770 (19.33)	1 (Ref)	
2 (+ 3–20%)	3986 (24.69)	3167 (79.45)	819 (20.55)	1.08 (0.97–1.20)	0.175
3 (+ 20–45%)	3981 (24.66)	2987 (75.03)	994 (24.97)	1.39 (1.25–1.54)	< 0.001
4 (+ 45–1091%)	3985 (24.68)	3141 (78.82)	844 (21.18)	1.12 (1.01–1.25)	0.040
5 (new herd)	211 (1.31)	129 (60.14)	82 (38.86)	2.65 (1.99–3.54)	< 0.001
Herd location					
1 (SE)	5122 (31.72)	4101 (80.07)	1021 (19.93)	1 (Ref)	
2 (SW)	7145 (44.25)	5684 (79.55)	1461 (20.45)	1.03 (0.94–1.12)	0.485
3 (NW)	1394 (8.63)	1108 (79.48)	286 (20.52)	1.04 (0.90–1.20)	0.630
4 (NE)	2485 (15.39)	1744 (70.18)	741 (29.82)	1.71 (1.53–1.91)	< 0.001
Herd Size					
1 (0–89)	4074 (25.23)	3415 (83.82)	659 (16.18)	1 (Ref)	
2 (90–139)	4001 (24.78)	3227 (80.65)	774 (19.35)	1.24 (1.09–1.36)	< 0.001
3 (140–213)	4057 (25.13)	3119 (76.88)	938 (23.12)	1.55 (1.39–1.74)	< 0.001
4 (214–2299)	4014 (24.86)	2876 (77.65)	1138 (28.35)	2.05 (1.84–2.29)	< 0.001
Mart Moves					
0 (no move)	2958 (18.32)	2289 (77.38)	669 (22.62)	1 (Ref)	
1 (Any move)	13,188 (81.68)	10,348 (78.47)	2840 (21.53)	0.94 (0.86–1.03)	0.197
Farm Moves					
0 (no move)	1340 (8.30)	1089 (81.27)	255 (18.73)	1 (Ref)	
1 (any move)	14,806 (91.70)	11,548 (78.00)	3258 (22.00)	1.22 (1.06–1.41)	0.005
All movements					
1 (0–11)	4233 (26.22)	3374 (79.71)	859 (20.29)	1 (Ref)	
2 (12–35)	3874 (23.99)	3071 (79.27)	803 (20.73)	1.03 (0.92–1.14)	0.628
3 (36–103)	4012 (24.85)	3207 (79.94)	805 (20.06)	0.99 (0.89–1.10)	0.796
4 (≥ 104)	4027 (24.94)	2985 (74.12)	1042 (25.88)	1.37 (1.24–1.52)	< 0.001
BVD status					
0 (negative)	15,599 (96.61)	12,238 (78.45)	3361 (21.55)	1 (Ref)	
1 (positive)	547 (3.39)	399 (72.94)	148 (27.06)	1.35 (1.11–1.64)	0.002

### Assessment of confounding and effect modification

Herd size caused a decrease in the strength of the association between herd expansion and mortality outcome. Consequently, herd size was selected as a confounder in the association between mortality outcome and herd expansion. The association between herd expansion and mortality outcome did not vary for other potential confounding variables, p-value test for homogeneity of OR’s > 0.05.

### Collinearity

Variables showing significant association (using the chi-squared test) with mortality outcomes were tested for collinearity. No variable was highly correlated to another, and Pearson’s correlation coefficients were all < 0.5.

### Multivariable logistic regression analysis

The final logistic regression model between herd expansion and calf mortality under 6 months is shown in Table 4. Manual backward stepwise logistic regression was performed while keeping herd size, a confounder, on top of the variables list. The selected covariates were added to the final logistic regression model. Additionally, likelihood ratio tests (LRT) were used to assess if herd size (an ordinal variable) should be included as a linear variable.

Herd location and herd size were retained in the final logistic regression model. All farm movements and herd BVD status were removed, as the association with mortality was insignificant (*p*-value > 0.05) after adjusting for other covariates.

**Table 4:** Multivariable logistic regression results showing adjusted odds ratios for the association between herd expansion, potential confounders and mortality in calves under 6 months

Risk Factor	Odds ratio	95% CI	*P* value
Calves under six months			
Herd expansion			
1 (same size)	1 (Ref)	1 (Ref)	
2 (small)	1.00	0.90–1.12	0.989
3 (moderate)	1.23	1.10–1.37	< 0.001
4 (large)	1.22	1.09–1.36	0.001
5 (new herd)	2.44	1.82–3.29	< 0.001
Herd location			
1 (SE)	1 (Ref)	1 (Ref)	
2 (SW)	1.20	1.09–1.31	< 0.001
3 (NW)	1.23	1.06–1.42	0.008
4 (NE)	1.91	1.71–2.14	< 0.001
Herd size	1.29	1.24–1.34	< 0.001

#### Herd expansion

The increased chance of high mortality remained significant in all expansion categories (level 3, 4, 5) after adjusting for covariates. Level 3 expansion category had a 23% (95% CI:10–37%) increase, level 4 had 22% (95% CI:9–36%%) increase, while newly established herds had 244% increase in odds of poorer mortality outcome compared to herds that did not increase in size (OR 2.44, 95% CI:1.82–3.30). Level 2 expansion category was not associated with poor herd calf mortality.

#### Herd location

Herds located in the north-east of the country were more likely to have high calf mortality, OR 1.70 (95% CI: 1.70–2.12, *p*-value < 0.001). Followed by herds in the north-west, OR 1.23 (95% CI: 1.06–1.42, *p*-value = 0.01), and south-west, OR 1.20 (95% CI: 1.09–1.31, *p*-value < 0.001) compared to herds in the south-east.

#### Herd size

A LRT for inclusion of herd size as a linear variable, yielded a p-value of 0.45, indicating no evidence against the null hypothesis of no linear association, thus its inclusion as a linear variable. There was a significant 29% increase in odds of high mortality per unit increase in herd size category (95% CI: 24–34%, *p*-value < 0.001).

### Sensitivity analysis

The logistic model for mortality in calves under 6 months correctly predicted the outcome in 78.27% of observations and the area under the curve (AUC) was 0.60.

## Discussion

### Statement of principal findings

Herd expansion greater than 20% over the five-year study period, was associated with higher herd mortality classification in calves under 6 months of age compared to herds that did not increase in size. Newly established herds were more likely to have poor mortality outcomes. Herd location and herd size were significantly associated with mortality outcome. Herd calf mortality was not affected by number of cattle movements onto the farm or herd BVD status.

### Strengths and weaknesses of the study

AIM data analysed in this study provides nationally representative statistics on all dairy herds in Ireland, so the results are generalizable. National cattle registers have great potential for monitoring trends in calf mortality and for exploring associations between relevant risk factors [15, 16, 24, 46]. However, as with all national level data, records may not be accurate or complete. There is a potential for exclusion of stillborn calves and calves that may have died before tagging/registration has taken place, which may lead to an underestimation of mortality rates in calves under 6 months. A study by Mee et al. [19], identified that 46% of herds had no record of perinatal mortality rate. However, since the introduction of the compulsory BVD eradication programme in Ireland, this percentage is likely reduced.

Within DAFM, calf mortality is expressed as the number of calves registered as dead by a specified age divided by the number of births. The selected ages are six weeks, six months and one year. While this provides a reasonable estimation, there are drawbacks. Herds that sell calves pass their risk to the buyer, as calves are no longer present on farm to run the risk of dying, the converse is also true for herds that purchase calves. However, this metric for calf survival allows comparisons with other countries that use a similar measure [24, 27].

### Meaning of the study: possible implications for policymakers

Approximately 80% of herds achieved calf mortality levels < 10% in calves under 6 months, which compares favourably to levels reported in other countries [23, 24, 31].

Reasons for higher levels of calf mortality in herds that have expanded are likely to be multifactorial. As herd size increases, stocking density increases which in turn increases potential for any infectious diseases to build up and spread [11]. Expansion in animal numbers also requires more labour as more animals need to be cared for. There is less time for individual animal attention which may lead to early signs of sickness or ill-health going unnoticed. If appropriate infrastructure is not in place to cater for any increase in animal numbers, then this may lead to increased deaths. Housing quality has also been found to be a risk factor for calf mortality [28, 33]. A study by Sinnott et al. [47], found that only a small percentage of Irish herdowners had updated animal housing and many had adapted older buildings to cope with increased animal numbers.

This study demonstrated significant regional differences in mortality levels of cattle up to six months, with the north-east of the country most likely to have high herd calf mortality, supporting previous findings by Lane [15, 16]. Reimus et al. [28] in Estonia and Gulliksen et al. [23] in Norway. The observed differences may be a result of distinct management factors, such as herd calving patterns i.e., dual season calving, with cows calving in spring and autumn, all year-round calving herds or other confounding local variables contributing to this effect. Previous studies have found season of birth, may be a risk factor for increased calf mortality, with more deaths occurring in winter than in spring [18] and [23].

Studies examining the effect of herd size on calf mortality have reported equivocal results. McAloon et al. [17], Lava et al. [26], Gulliksen et al. [23], demonstrated mortality risk increased with increasing herd size, while Barry et al. [14] and Zucali et al. [31], found no association between herd size and mortality risk. This study demonstrated a linear association between herd size and mortality level. Larger herd sizes contribute to less individual animal attention, and which may lead to increased mortality rates [40].

Purchase of animals has long been associated with increased mortality risk as herd biosecurity is breached with potential for introduction of diseases into the herd [27, 30, 41]. Results of the present study did not find an association between animal movements onto the farm and herd calf mortality level in animals aged under 6 months. This may be explained by the length of time at risk or differences in age classes of animals bought into the herd with lack of exposure of younger animals to purchased animals. Dairy herds are also more likely to move animals off the farm, through the sale of calves rather than move animals in [48]. This rationale might also explain why no association between herd mortality level and herd BVD status was found. 

The results of the present study have practical applications for policymakers and could inform targeted calf health and welfare inspections and infectious disease surveillance systems. As highlighted in previous studies [49] calf mortality alone cannot not be used as a welfare indicator but should be interpreted with other variables. Focussed on-farm investigations will help to identify individual farm level variables that may be responsible for mortality in high mortality herds so timely decision making and recommendations can be made to improve calf rearing outcomes.

National registry data will be further improved following the recent role out of the national veterinary prescription system in Ireland. This online platform will allow real time recording of all prescription-based medicines in cattle as is the situation in Finland [29]. Failure to maintain diagnostic and treatment records are deleterious to herd disease problem solving and welfare outcomes.

### Unanswered questions and future research

Mortality monitoring may lack the timeliness to be used as an early indicator of unexpected health events [46] unless carried out at regular intervals. Standardised mortality analysis as proposed by Pannwitz [42] could be used to predict the number of deaths in the national herd based on a certain number of sampled holdings, allowing high counts to be followed up as appropriate. This may also act as an alert system for targeted herd investigations and is an area that warrants further investigation.

Enrolling herds at risk of high mortality in a study, similar to Svensson et al. [40], to submit animals that die for necropsy examination in state laboratories, would help to identify individual farm level factors responsible for calf mortality. A study by Kennedy et al. [50] indicated that submission of animals to state laboratories in Ireland contributes to promotion of animal health and welfare, through implementation of vaccination programs and other management changes.

### Limitations

The primary limitation of this cross-sectional analytical study is lack of temporality in the association between herd expansion and mortality level as both are assessed at the same time, so there is limited ability to confirm causal relationships. Furthermore, the analysis in the present study represents herd level data, individual or calf level data on causes of mortality are not available from national database records, consequently, the results of the present study cannot be extrapolated to the individual level, to avoid ecological fallacy.

Fixed effects logistic regression modelling was used in the present study. This may not account for within herd and between herd variation in risk factors for calf mortality. However, dichotomizing the main outcome variable (mortality) and analysing data over a five-year period, may help minimise this variation.

### Conclusions and recommendations

This study has demonstrated that herds that have expanded > 20% and newly established are more likely to have high herd calf mortality. It highlights herds that may benefit from targeted emphasis on herd health management.

Calf mortality levels in the present study, equate favourable to those reported in other countries with predominately grass based dairy enterprises [24, 37]. This study also demonstrates the value of analysis of national data as a tool to determine optimisation of farm investigations and surveillance and policy decisions to prioritise animal health and welfare. The identification of risk factors for calf mortality can allow for targeted education, interventions, further research, and preventative measures. Industry wide data analysis on this scale is fundamental to providing initial benchmark estimates for dairy farmers and their advisors, it also provides transparency to all stakeholders. Furthermore, it can be used to measure the success of any interventions made. National Data can be used to optimise national herd health and welfare and make the future of dairy farming sustainable.

## Data Availability

The datasets analysed in the study are available from the data controller DAFM on reasonable request and subject to anonymisation of any personal data compliant with GDPR. For data requests please contact the first author.

## Abbreviations

DAFM: Department of agriculture, food and the marine
AIM: Animal identification and movement
BVD: Bovine viral diarrhoea
LRT: Likelihood ratio test
ROC: Receiver operating characteristic
AUC: Area under curve
